# Non contiguous-finished genome sequence and description of *Cellulomonas massiliensis* sp. nov.

**DOI:** 10.4056/sigs.3316719

**Published:** 2012-12-15

**Authors:** Jean-Christophe Lagier, Dhamodharan Ramasamy, Romain Rivet, Didier Raoult, Pierre-Edouard Fournier

**Affiliations:** 1 Aix-Marseille Université, Faculté de médecine, Marseille, France

**Keywords:** *Cellulomonas massiliensis*, genome

## Abstract

*Cellulomonas massiliensis* strain JC225^T^ sp. nov. is the type strain of *Cellulomonas massiliensis* sp., a new species within the genus *Cellulomonas*. This strain, whose genome is described here, was isolated from the fecal flora of a healthy Senegalese patient. *C. massiliensis* is an aerobic rod-shaped bacterium. Here we describe the features of this organism, together with the complete genome sequence and annotation. The 3,407,283 bp long genome contains 3,083 protein-coding and 48 RNA genes.

## Introduction

*Cellulomonas massiliensis* strain JC225^T^ (= CSUR P160 = DSM 25695) is the type strain of *C. massiliensis* sp. nov. This bacterium is a motile, Gram-positive, aerobic, indole-negative rod that was isolated from the stool of a healthy Senegalese patient as part of a culturomics study aiming at cultivating all bacterial species within human feces [1].

The current approach to the classification of prokaryotes, known as polyphasic taxonomy, relies on a combination of phenotypic and genotypic characteristics [2]. However, as more than 3,000 bacterial genomes have been sequenced [3], and proteomic information is more becoming more readily accessible [4], we recently proposed that genomic information should be integrated in the description of new bacterial species [5-11].

The genus *Cellulomonas* was created in 1923 to reclassify several bacteria previously classified as *Bacillus* species [12]. To date, this genus is made of 19 species [13-24]. The two species that are the most phylogenetically related to *C. massiliensis* are *C. composti* [17] and *C. persica* [21]. Most of these species were originally solated from environmental samples, notably from habitats enriched in cellulose, such as soil or sugar fields, and occasionally from the rumen and activated sludge. Rare cases of human endocarditis [25], osteomyelitis [25], endophtalmitis [26] and cholecystitis [27] caused by *Cellulomonas* species have been reported. To date, members of the genus *Cellulomonas* have not been described in the normal fecal flora.

Here we present a summary classification and a set of features for *C. massiliensis* sp. nov. strain JC225^T^ together with the description of the complete genomic sequencing and annotation. These characteristics support the circumscription of the species *C. massiliensis.*

## Classification and features

A stool sample was collected from a healthy 16-year-old male Senegalese volunteer patient living in Dielmo (a rural village in the Guinean-Sudanian zone in Senegal), who was included in a research protocol. Written assent was obtained from this individual; no written consent was needed from his guardians for this study because he was older than 15 years old (in accordance with the previous project approved by the Ministry of Health of Senegal and the assembled village population and as published elsewhere [28].

**Figure 1:**
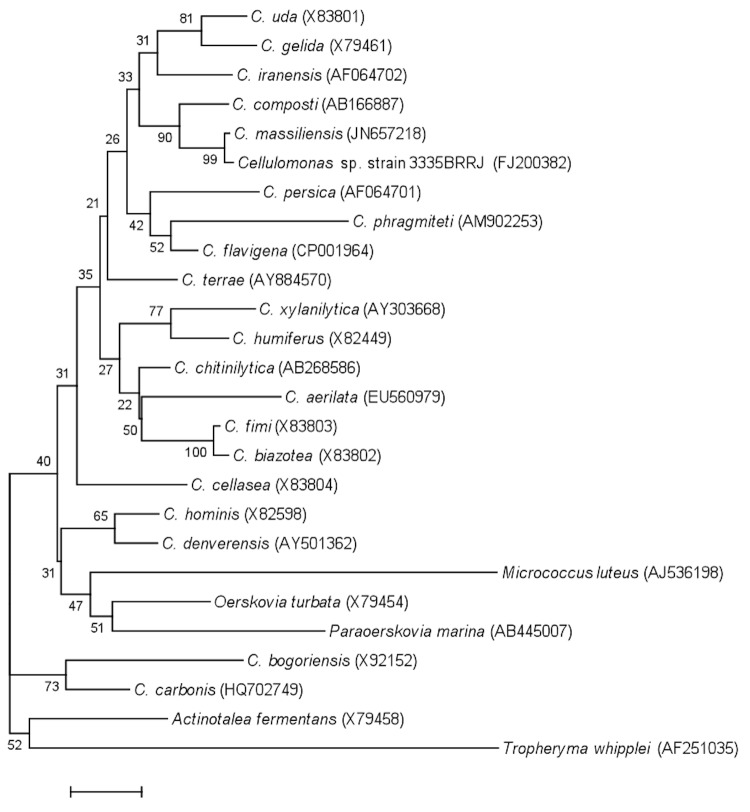
Phylogenetic tree highlighting the position of *Cellulomonas massiliensis* strain JC225^T^ relative to other type strains within the genus *Cellulomonas* and other members of the family *Cellulomonadaceae*. GenBank accession numbers are indicated in parentheses. Sequences were aligned using CLUSTALW, and phylogenetic inferences obtained using the maximum-likelihood method within the MEGA software. Numbers at the nodes are bootstrap values obtained by repeating the analysis 500 times to generate a majority consensus tree. The scale bar indicates a 1% nucleotide sequence divergence.

Both this study and the assent procedure were approved by the National Ethics Committee of Senegal (CNERS) and the Ethics Committee of the Institut Fédératif de Recherche IFR48, Faculty of Medicine, Marseille, France (agreement numbers 09-022 and 11-017). Several other new bacterial species were isolated from this specimen using various culture conditions, including the recently described *Anaerococcus senegalensis*, *Bacillus timonensis*, *Alistipes senegalensis*, *Alistipes timonensis*,*Clostridium senegalense*, *Paenibacillus senegalensis* and *Peptoniphilus timonensis* [5-11], thus suggesting that the human digestive flora is far from being fully known. The fecal specimen was preserved at -80°C after collection and sent to Marseille. Strain JC225 (Table 1) was isolated in May 2011 by passive filtration of the stool and aerobic incubation on Brain Heart Infusion agar at 37°C. This strain exhibited a nucleotide sequence similarity of 98.3% with *Cellulomonas composti* (Kang *et al* 2007), the phylogenetically closest validated *Cellulomonas* species (Figure 1) that was cultivated from cattle farm compost [17]. This value was lower than the 98.7% 16S rRNA gene sequence threshold recommended by Stackebrandt and Ebers to delineate a new species without carrying out DNA-DNA hybridization [39]. By comparison to the Genbank database [40] strain JC225^T^ also exhibited a nucleotide sequence similarity greater than 99.5% with *Cellulomonas* sp. strain 3335BRRJ isolated from clean room environments (Genbank accession number FJ200382). This bacterium is most likely classified within the same species as strain JC225 ^T^ (Figure 1).

**Table 1:** Classification and general features of *Cellulomonas massiliensis* strain JC225^T^

**MIGS ID**	**Property**	**Term**	**Evidence code^a^**
	Current classification	Domain *Bacteria*	TAS [29]
		Phylum *Actinobacteria*	TAS [30]
		Class *Actinobacteria*	TAS [31]
		Order *Actinomycetales*	TAS [31-34]
		Family *Cellulomonadaceae*	TAS [31,34-37]
		Genus *Cellulomonas*	TAS [12,32]
		Species *Cellulomonas massiliensis*	IDA
		Type strain JC225^T^	IDA
	Gram stain	positive	IDA
	Cell shape	rod	IDA
	Motility	positive	IDA
	Sporulation	nonsporulating	IDA
	Temperature range	mesophilic	IDA
	Optimum temperature	37°C	IDA
MIGS-6.3	Salinity	growth in BHI medium + 5% NaCl	IDA
MIGS-22	Oxygen requirement	aerobic	IDA
	Carbon source	galactose	NAS
	Energy source	chemoorganotrophic	NAS
MIGS-6	Habitat	human gut	IDA
MIGS-15	Biotic relationship	free living	IDA
MIGS-14	Pathogenicity Biosafety level Isolation	unknown 2 human feces	NAS
MIGS-4	Geographic location	Senegal	IDA
MIGS-5	Sample collection time	September 2010	IDA
MIGS-4.1	Latitude	13.7167	IDA
MIGS-4.1	Longitude	– 16.4167	IDA
MIGS-4.3	Depth	surface	IDA
MIGS-4.4	Altitude	51 m above sea level	IDA

Evidence codes - IDA: Inferred from Direct Assay; TAS: Traceable Author Statement (i.e., a direct report exists in the literature); NAS: Non-traceable Author Statement (i.e., not directly observed for the living, isolated sample, but based on a generally accepted property for the species, or anecdotal evidence). These evidence codes are from the Gene Ontology project [38]. If the evidence is IDA, then the property was directly observed for a live isolate by one of the authors or an expert mentioned in the acknowledgements.

**Figure 2:**
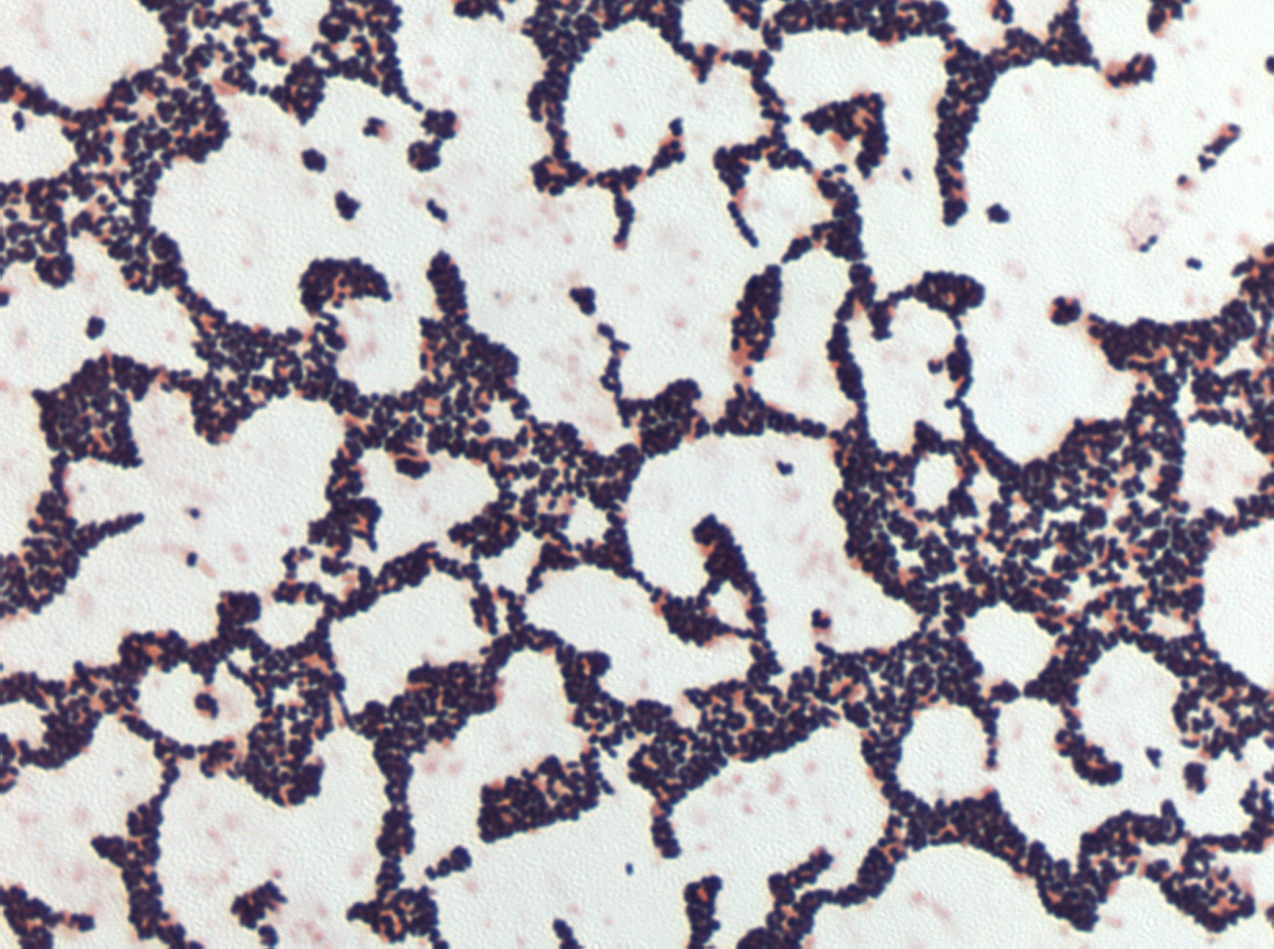
Gram staining of *C. massiliensis* strain JC225^T^

**Figure 3:**
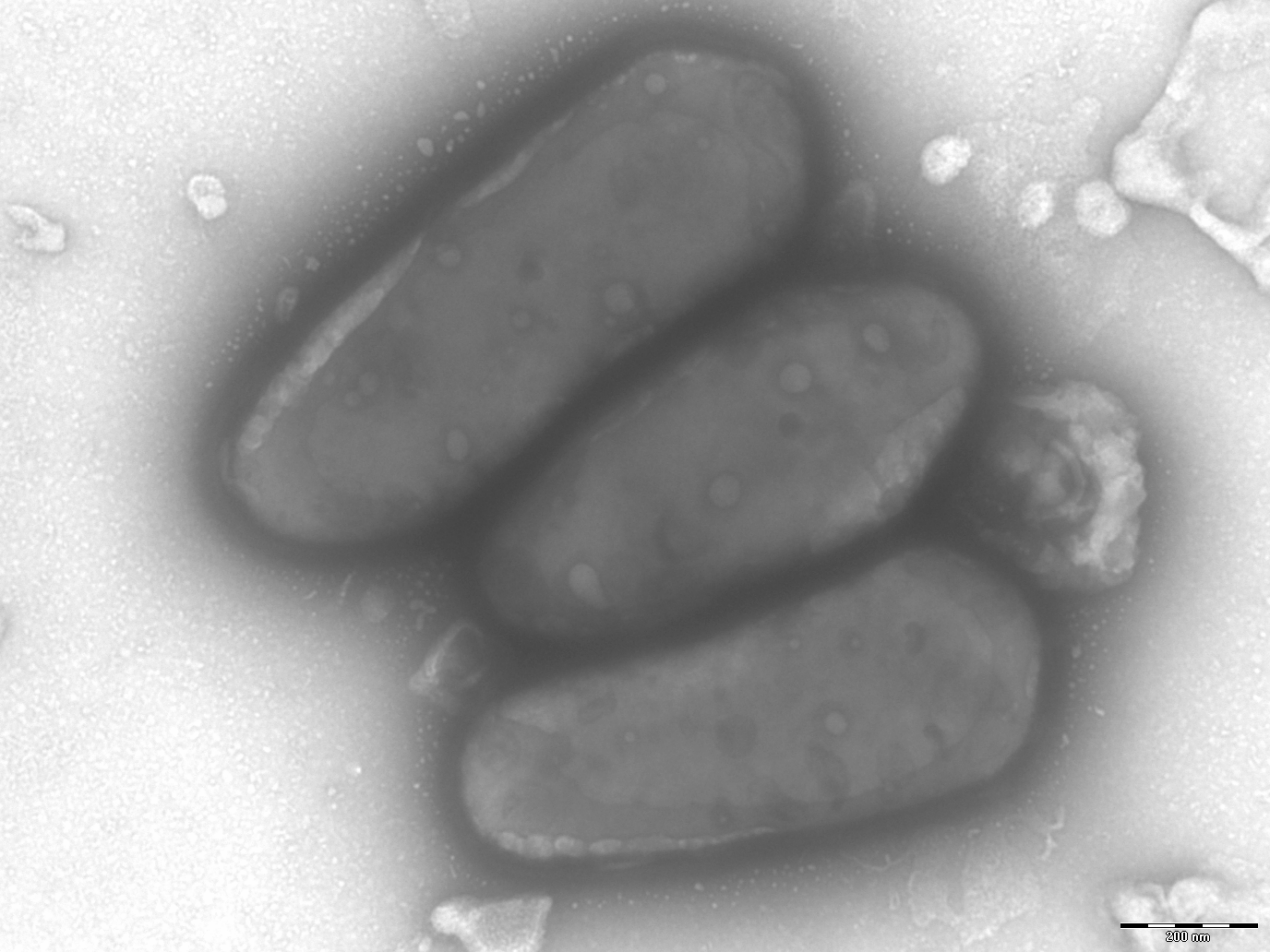
Transmission electron microscopy of *C. massiliensis* strain JC225^T^, using a Morgani 268D (Philips) at an operating voltage of 60kV. The scale bar represents 200 nm.

Different growth temperatures (25, 30, 37, 45°C) were tested; no growth occurred at 25°C or 45°C, growth occurred between 30 and 37°C, and optimal growth was observed at 37°C. Colonies were transparent and smooth with a diameter of 1 mm on blood-enriched Columbia agar and Brain Heart Infusion (BHI) agar. Growth of the strain was tested under anaerobic and microaerophilic conditions using GENbag anaer and GENbag microaer systems, respectively (BioMérieux), and in the presence of air, with or without 5% CO_2_. Optimal growth was achieved aerobically. Weak growth was observed under microaerophilic condition and with 5% CO_2._ No growth was observed under anaerobic conditions. Gram staining showed Gram-positive rods. A motility test was positive. Cells grown on agar are Gram-positive (Figure 2), with a diameter and length ranging from 0.37 to 0.60 µm (mean, 0.48 µm), and from 0.55 to 1.4 µm (mean, 0.95 µm), respectively, in electron microscopy, (Figure 3).

Strain JC225^T^ exhibited catalase and oxidase activities. Using the API 20 NE system (BioMérieux), a positive reaction was obtained for aesculin hydrolysis and β-galactosidase. Negative reactions were obtained for nitrate reduction, indole production, glucose fermentation, arginine dihydrolase, urease, gelatin hydrolysis, and glucose, arabinose, mannose, mannitol N-acetyl-glucosamine, maltose, gluconate, caprate, adipate, malate, citrate, and phenyl-acetate assimilation. *C. massiliensis* is susceptible to amoxicillin, imipenem, gentamicin, and ciprofloxacin but resistant to trimethoprim/sulfamethoxazole and metronidazole. By comparison to *C. composti* [17], *C. massiliensis* differed in motility, nitrate reduction, gelatine hydrolysis, carbohydrate assimilation, and catalase activity (Table 2).

**Table 2:** Differential phenotypic characteristics of five *Cellulomonas* strains^†^.

Properties	*C. massiliensis*	*C. composti*	*C. persica*	*C. flavigena*	*C. iranensis*
Oxygen requirement	aerobic	Facultative anaerobic	aerobic	aerobic	aerobic
Gram stain	+	+	+	+	+
Motility	+	–	+	–	+
**Production of**					
Catalase	+	–	na	+	na
Nitrate reductase	–	+	+	+	+
Urease	–	–	+	-	+
β-galactosidase	+	na	na	na	na
N-acetyl-glucosamine	–	na	na	na	na
Arginine dihydrolase	–	na	na	na	na
**Fermentation for**					
Sucrose	–	+	na	+	na
Glucose	–	+	na	+	na
Mannitol	–	–	na	–	na
Gluconate	–	–	na	W	na
Maltose	–	+	na	+	na
**Hydrolysis of**					+
Gelatin	+	w	w	+	w
Esculin	–	+	na	+	na
**G+C content (mol%)**	71.2	73.7	na	72.7-74.8	na
**Habitat**	Human gut	Compost	Forest humus soil	Soil	Forest humus soil

^†^*Cellulomonas massiliensis strain* JC225^T^, *Cellulomonas composti* strain TR7-06 ^T^ , *Cellulomonas persica* strain I^T^, *Cellulomonas flavigena* strain DSM 20109 ^T^, and *Cellulomonas iranensis* strain O^T^.

**Figure 4:**
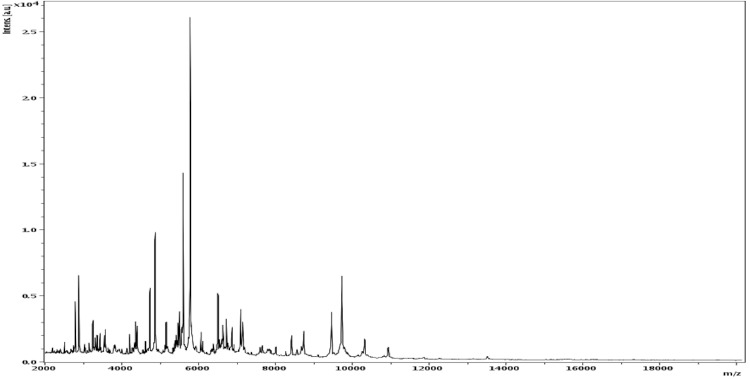
Reference mass spectrum from *C. massiliensis* strain JC225^T^. Spectra from 12 individual colonies were compared and a reference spectrum was generated.

na = data not available; w = weak,

na = data not available; w = weak,

Matrix-assisted laser-desorption/ionization time-of-flight (MALDI-TOF) MS protein analysis was carried out as previously described [5,41] using a Microflex spectrometer (Bruker Daltonics, Germany). Twelve distinct deposits were done for strain JC225 from 12 isolated colonies. The 12 JC225 spectra were imported into the MALDI BioTyper software (version 2.0, Bruker) and analyzed by standard pattern matching (with default parameter settings) against the main spectra of 3,769 bacteria, which were used as reference data in the BioTyper database. The database contained 11 spectra from 8 validly published *Cellulomonas* species, including *Cellulomonas composti,* the phylogenetically closest species to *C. *massiliensis. No significant score was obtained for strain JC225^T^, thus suggesting that our isolate was not a member of a known species within the Bruker database. We incremented our database with the reference spectrum from strain JC225^T^ (Figure 4).

## Genome sequencing information

### Genome project history

The organism was selected for sequencing on the basis of its phenotypic differences, phylogenetic position and 16S rRNA similarity to other members of the genus *Cellulomonas* and is part of a study of the human digestive flora aiming at isolating all bacterial species within human feces. It was the fourth genome of a *Cellulomonas* species and the first genome of *Cellulomonas massiliensis* sp. nov. The EMBL accession number is CAHD00000000 and consists of 250 contigs (>=200 bp). Table 3 shows the project information and its association with MIGS version 2.0 compliance [42].

**Table 3:** Project information

**MIGS ID**	**Property**	**Term**
MIGS-31	Finishing quality	High-quality draft
MIGS-28	Libraries used	Paired-end 3 Kb library
MIGS-29	Sequencing platforms	454 GS FLX Titanium
MIGS-31.2	Fold coverage	25×
MIGS-30	Assemblers	Newbler version 2.5.3
MIGS-32	Gene calling method	Prodigal
	EMBL ID	CAHD00000000
	EMBL Date of Release	May 30, 2012
	Project relevance	Study of the human gut microbiome

### Growth conditions and DNA isolation

*Cellumonas massiliensis* sp. nov. JC225^T^ (= CSUR P160 = DSM 25695) was grown aerobically on 5% sheep blood-enriched Columbia agar (BioMérieux) at 37°C. Ten petri dishes were spread and resuspended in 3×100µl of G2 buffer (EZ 1 DNA Tissue kit, Qiagen). A first mechanical lysis was performed using glass powder on a Fastprep-24 device (MP Biomedicals, Ilkirch, France) during 2×20 seconds. DNA was then treated with 2.5µg/µL lysozyme (30 minutes at 37°C) and extracted using a BioRobot EZ 1 Advanced XL (Qiagen). The DNA was then concentrated and purified using a Qiamp kit (Qiagen). The yield and the concentration were measured using a Quant-it Picogreen kit (Invitrogen) on a Genios_Tecan fluorometer at 78.9 ng/µl.

### Genome sequencing and assembly

Both shotgun sequencing and paired-end sequencing strategies were used (Roche). Both libraries were pyrosequenced on a GS FLX Titanium sequencer (Roche). This project was loaded onto a single 1/4 region of a PTP Picotiterplate (Roche, Meylan, France) for the shotgun library and 2 ×1/4 region for the 3-kb paired-end library. The shotgun library was constructed with 500ng of DNA with the GS Rapid library Prep kit as described by the manufacturer (Roche). For the paired-end library, 5µg of DNA was mechanically fragmented on a Hydroshear device (Digilab, Holliston, MA, USA) with an enrichment size at 3-4kb. The DNA fragmentation was visualized through an Agilent 2100 BioAnalyzer on a DNA labchip 7500 with an optimal size of 3.216 kb. The library was constructed according to the 454 Titanium paired-end protocol (Roche). Circularization and nebulization were performed and generated a pattern with an optimum at 395 bp. After PCR amplification through 17 cycles followed by double size selection, the single stranded paired-end library was quantified on with a Quant-it Ribogreen kit (Invitrogen) on a Genios Tecan fluorometer at 132pg/µL. The library concentration equivalence was calculated at 6.11E+08 molecules/µL. The libraries were stored at -20°C until further use.

The shotgun library was clonally amplified with 3 cpb in 3 emPCR reactions and the 3-kb paired-end library was amplified with 0.5cpb in 4 emPCR reactions with the GS Titanium SV emPCR Kit (Lib-L) v2 (Roche). The yield of the shotgun emPCR reactions was 10.13%, and the yields of the paired-end emPCRs was 8.6%, in the range of 5 to 20% from the Roche procedure.

Approximately 790,000 beads for both the shotgun and paired-end libraries were loaded on the GS Titanium PicoTiterPlate PTP Kit 70×75 and sequenced with the GS FLX Titanium Sequencing Kit XLR70 (Roche). The runs were performed overnight and then analyzed on the cluster through the gsRunBrowser and Newbler Assembler (Roche). A total of 255,758 and 256,082 passed filter wells were obtained for the shotgun and paired-end strategies, respectively, and generated 86.75 and 78.45 Mb of DNA sequence with length averages of 339 and 313 bp, respectively. The filter passed sequences were assembled using Newbler with 90% identity and 40 bp as overlap. The final assembly identified 250 contigs (>200 bp) arranged into 5 scaffolds and generated a genome size of 3.40 Mb.

### Genome annotation

Open Reading Frames (ORFs) were predicted using Prodigal [43] with default parameters but the predicted ORFs were excluded if they were spanned a sequencing GAP region. The predicted bacterial protein sequences were searched against the GenBank database [40] and the Clusters of Orthologous Groups (COG) databases using BLASTP. The tRNAscan-SE tool [44] was used to find tRNA genes, whereas ribosomal RNAs were found by using RNAmmer [45]. Transmembrane domains and signal peptides were predicted using TMHMM [46] and SignalP [47], respectively. ORFans were identified if their BLASTp *E-*value was lower than 1e-03 for alignment length greater than 80 amino acids. If alignment lengths were smaller than 80 amino acids, we used an *E*-value of 1e-05. Such parameter thresholds have been used in previous works to define ORFans. To estimate the mean level of nucleotide sequence similarity at the genome level between *C. massiliensis* and *C. flavigena* and *C. fimi* (EMBL accession numbers CP001964 and CP002666, respectively), the only two available genomes from validly published *Cellulomonas* species to date, we compared the ORFs only using BLASTN at a query coverage of ≥ 70% and a minimum nucleotide length of 100 bp.

## Genome properties

**Figure 5:**
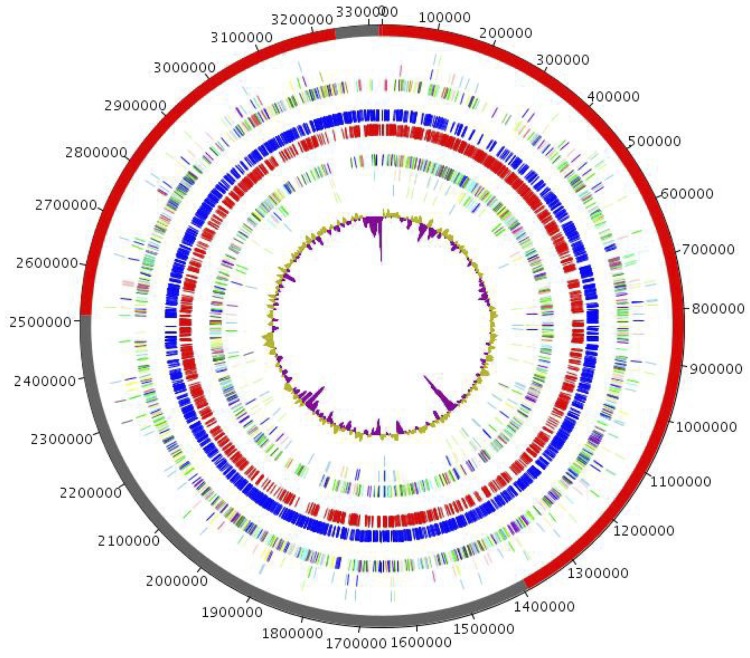
Graphical circular map of the *C. massiliensis* strain JC225^T^ genome. From outside to the center: scaffolds (red / grey), COG category of genes on the forward strand (three circles), genes on forward strand (blue circle), genes on the reverse strand (red circle), COG category on the reverse strand (three circles), G+C content.

The genome is 3,407,283 bp long (1 chromosome, but no plasmid) with a 71.22% G+C content (Table 4 and Figure 5). It is composed of 5 scaffolds. Of the 3,131 predicted genes, 3,083 were protein-coding genes, and 48 were RNAs (1 rRNA operon and 45 tRNA genes). A total of 2,184 genes (70.84%) were assigned a putative function, and 256 genes were identified as ORFans (8.30%). The remaining genes were annotated as hypothetical proteins. The distribution of genes into COGs functional categories is presented in Table 5. The properties and the statistics of the genome are summarized in Table 4 and 5.

**Table 4:** Nucleotide content and gene count levels of the genome

**Attribute**	**Value**	**% of total^a^**
Genome size (bp)	3,407,283	100
DNA coding region (bp)	3,091,035	90.72
DNA G+C content (bp)	2,426,733	71.22
Total genes	3,131	100
RNA genes	48	1.53
Protein-coding genes	3,083	98.47
Genes with function prediction	2,184	70.84
Genes assigned to COGs	2,155	69.9
Genes with peptide signals	387	12.55
Genes with transmembrane helices	721	23.39

The total is based on either the size of the genome in base pairs or the total number of protein coding genes in the annotated genome.

**Table 5:** Number of genes associated with the 25 general COG functional categories

**Code**	**Value**	**%age**	**Description**
J	140	4.54	Translation
A	1	0.03	RNA processing and modification
K	225	7.3	Transcription
L	110	3.57	Replication, recombination and repair
B	1	0.03	Chromatin structure and dynamics
D	20	0.65	Cell cycle control, mitosis and meiosis
Y	0	0	Nuclear structure
V	27	0.88	Defense mechanisms
T	116	3.76	Signal transduction mechanisms
M	111	3.6	Cell wall/membrane biogenesis
N	42	1.36	Cell motility
Z	0	0	Cytoskeleton
W	1	0.03	Extracellular structures
U	41	1.33	Intracellular trafficking and secretion
O	82	2.66	Posttranslational modification, protein turnover, chaperones
C	132	4.28	Energy production and conversion
G	253	8.21	Carbohydrate transport and metabolism
E	276	8.95	Amino acid transport and metabolism
F	68	2.21	Nucleotide transport and metabolism
H	85	2.76	Coenzyme transport and metabolism
I	68	2.21	Lipid transport and metabolism
P	112	3.63	Inorganic ion transport and metabolism
Q	42	1.36	Secondary metabolites biosynthesis, transport and catabolism
R	323	10.48	General function prediction only
S	175	5.68	Function unknown
-	928	30.1	Not in COGs

The total is based on the total number of protein coding genes in the annotated genome.

### Comparison with the genomes from other *Cellulomonas* species

Here, we compared the genome sequence of *C. massiliensis* strain JC225^T^ with those of *C. flavigena* strain 134^T^ [48] and *C. fimi* strain ATCC 484^T^ (EMBL accession number CP002666). The draft genome sequence of *C. massiliensis* has a smaller size than those of *C. flavigena* and *C. fimi* (3.40 *vs* 4.12 and 4.26 Mb, respectively), a lower G+C content (71.22 *vs* 74.3 and 74.7, respectively), and a smaller number of predicted genes (3,131 vs 3,788 and 3,863, respectively). In addition, *C. massiliensis* shared a mean 88.75% (range 70.01-100%) and 89.61% (range 70.07-100%) sequence similarity with *C. flavigena* and *C. fimi*, respectively, at the genome level.

## Conclusion

On the basis of phenotypic, phylogenetic and genomic analyses, we formally propose the creation of *Cellulomonas massiliensis* sp. nov. that contains the strain JC225^T^. This bacterium has been found in Senegal.

### Description of *Cellulomonas massiliensis* sp. nov.

*Cellulomonas massiliensis* (ma.si.li.e′n.sis. L. gen. masc. n. *massiliensis*, of Massilia, the Latin name of Marseille where was isolated *C. massiliensis*).

Colonies are transparent and smooth with a diameter of 1 mm on blood-enriched Columbia agar and Brain Heart Infusion (BHI) agar. Cells are rod-shaped with a diameter and length ranging from 0.37 to 0.60 µm (mean of 0.48 µm), and from 0.55 to 1.4 µm (mean of 0.95 µm), respectively. Optimal growth is achieved aerobically. Weak growth is observed with 5% CO_2_ and under microaerophilic conditions. No growth is observed under anaerobic conditions. Growth occurs between 30-37°C, with optimal growth at 37°C. Cells stain Gram-positive, are non-endospore forming, and are motile. Catalase, oxidase, aesculin hydrolysis and β-galactosidase activities are present. Indole production, nitrate reduction, glucose fermentation, arginine dihydrolase, urease, gelatin hydrolysis, and glucose, arabinose, mannose, mannitol N-acetyl-glucosamine, maltose, gluconate, caprate, adipate, malate, citrate, and phenyl-acetate assimilation activities are absent. Cells are susceptible to amoxicillin, imipenem, ciprofloxacin and gentamicin, but resistant to trimethoprim/sulfamethoxazole and metronidazole. The 16S rRNA and genome sequences are deposited in Genbank and EMBL under accession numbers JN657218 and CAHD00000000, respectively. The G+C content of the genome is 71.22%. The type strain JC225^T^ (= CSUR P160 = DSM 25695) was isolated from the fecal flora of a healthy patient in Senegal.
